# Phytochemical Analysis and Binding Interaction of Cotton Seed Cake Derived Compounds with Target Protein of *Meloidogyne incognita* for Nematicidal Evaluation

**DOI:** 10.3390/life12122109

**Published:** 2022-12-15

**Authors:** Fahad M. Almutairi, Amir Khan, Mohammad Rehan Ajmal, Rizwan Hasan Khan, Mohd Farhan Khan, Hira Lal, Mohammad Fahad Ullah, Faheem Ahmad, Lukman Ahamad, Arshad Khan, Hussain Arif, M. Ayaz Ahmad

**Affiliations:** 1 Physical Biochemistry Research Laboratory, Biochemistry Department, Faculty of Science, University of Tabuk, Tabuk 71491, Saudi Arabia; 2 Department of Botany, Aligarh Muslim University, Aligarh 202002, India; 3 Interdisciplinary Biotechnology Unit, Aligarh Muslim University, Aligarh 202002, India; 4 Department of Science, Gagan College of Management and Technology, Aligarh 202002, India; 5 Department of Chemistry, Aligarh Muslim University, Aligarh 202002, India; 6 Department of Medical Laboratory Technology, Faculty of Applied Medical Sciences, University of Tabuk, Tabuk 47512, Saudi Arabia; 7 Department of Biochemistry, Mohammad Ali Jauhar University, Rampur 244901, India; 8 Department of Physics, University of Tabuk, Tabuk 71491, Saudi Arabia

**Keywords:** phytochemical analysis, molecular docking, oil seed cake, plant-parasitic nematodes, sustainable management

## Abstract

The root-knot nematode *Meloidogyne incognita* is one of the most damaging plant-parasitic nematodes and is responsible for significant crop losses worldwide. Rising human health and environmental concerns have led to the withdrawal of commonly used chemical nematicides. There has been a tremendous demand for eco-friendly bio-nematicides with beneficial properties to the nematode hosting plants, which encourages the need for alternative nematode management practices. The current study was undertaken to determine the nematicidal potential of cotton seed cake (CSC) against second-stage juvenile (J2) hatching, J2 mortality, and J2 penetration of *M. incognita* in tomato plants in vitro. J2s and egg masses of *M. incognita* were exposed to four concentrations (250, 500, 750, and 1000 mg/L) of CSC extracts. The higher J2 mortality and inhibition of J2 hatching were found at 1000 mg/L, while the least effective result was observed at 250 mg/L of the CSC extract. The CSC extract applied with the concentrations mentioned above also showed inhibition of J2 penetration in tomato roots; 1000 mg/L showed the highest inhibition of penetration, while 250 mg/L displayed the least inhibition. Using gas chromatography-mass spectroscopy, we identified 11 compounds, out of which 9,12-Octadecadienoic acid, Hexadecanoic acid, and Tetradecanoic acid were found as major compounds. Subsequently, in silico molecular docking was conducted to confirm the nematicidal behavior of CSC based on binding interactions of the above three major compounds with the targeted protein acetylcholine esterase (AChE) of *M. incognita*. The values of binding free energy are −5.3, −4.5, and −4.9 kcal/mol, observed for 9,12-Octadecadienoic acid, n-Hexadecanoic acid, and Tetradecanoic acid, respectively, suggesting that 9,12-Octadecadienoic acid binds with the receptor AChE more efficiently than the other two ligands. This study indicates that CSC has nematicidal potential that can be used to control *M. incognita* for sustainable agriculture.

## 1. Introduction

Plant-parasitic nematodes (PPNs), particularly the root-knot species of the genus *Meloidogyne*, cause substantial yield losses on a large spectrum of horticultural and ornamental crops in all tropical and subtropical regions of the world [1]. Crop losses are attributable to phyto-nematodes at around 12.3% of the total world agricultural production annually, corresponding to an economic value of USD 157 billion [2]. *Meloidogyne incognita* is a sedentary endoparasite nematode affecting many plant species, including legumes and vegetables. They attack the roots of plants at the elongation region and shift to the vascular cylinder area where they start the formation of galls, which results in the deformation of vascular tissues [3]. Giant cells (GCs) surrounding the nematode’s head derive from parenchymatic stele cells, and their development initiates with a sequence of mitotic events not followed by cytokinesis, leading to the formation of multinucleated cells [4]. Following several rounds of nuclear divisions, GCs become hypertrophied, increasing in size [4]. Root-knot nematodes (RKNs) inhibit host plant development and interfere with nodulation and nitrogen fixation [3]. RKNs (*Meloidogyne* sp.) cause considerable damage to more than 5000 plant species and use their stylets to feed on the roots of the plants [5].

Chemical nematicides hugely impact PPNs; these also pose serious concerns regarding human health and the environment in developing countries. Therefore, with the pre-requisite for protecting human health and the environment, the search for novel alternative strategies is needed to manage the PPNs in the agro-ecosystem. Organic farming systems are typically more sustainable than conventional systems [6], and two global meta-analyses have shown that organic farming positively affects soil biota [7,8]. The impact of organic farming on soil nematode communities may depend upon the spatial scale, because nematode abundance and community composition can be related to edaphic and climatic variations across scales [9]. Oil seed cakes can be utilized as a source of organic fertilizers, since they are high in cellulose, hemicellulose, and lignin and have high nitrogen, phosphorus, and potassium [10]. Using non-edible oil seed cakes mainly protects plants from soil-borne phytopathogens, nematodes, and insect pests [11]. Oil cakes made from seeds such as castor beans and tung nuts are toxic to various detrimental microorganisms and exploited as fertilizers. In recent decades, oil seed cakes have been integrated with soil as organic amendments to improve crop yield and fertility. Various studies reported that these cakes adversely affect PPNs [12,13]. Several compounds, viz., saponins and volatile compounds (mainly 4-methylphenol) found in camellia seed cake, show nematicidal activity against *M. javanica* [14]. A byproduct of seed pressing known as seed cake is a rich source of lignocellulosic elements, proteins, amino acids, and lipids [15]. The release of various kinds of nitrogen and the stimulation of microbial activity in the soil are the key factors contributing to seed cakes’ antagonistic effects on nematodes [16].

On the other hand, oil seed cakes of neem, madhuca, and simarouba also possess antimicrobial compounds, found through GC-MS analysis, against broad-range pathogens [17]. However, GC-MS analysis from castor bean cake detected some volatile compounds. Among them, γ-decalactone exhibited good nematicidal potential against the RKN *M. incognita* [18]. Various organic matter of plant origin, including oil seed cakes, plant parts, and plant extracts, have been utilized as eco-friendly alternatives to control RKNs [19,20]. Radwan et al. reported that applied oil cakes containing 5, 10, 15, 20, or 50 g/kg soil of cotton, flax, olive, sesame, and soybean enhanced the growth of the plant, in addition to that populations of *M. incognita* in soil and root galling were significantly suppressed [21]. Goswami et al. reported in their study that the application of *Paecilomyces lilacinus* and *Trichoderma viride* alone or in combination with mustard cake and furadan promoted the growth of tomato plants and reduced the number of galls/plant, egg masses/root system, and eggs/egg mass [22]. Application of some oil cakes of neem, mahua, karanja, mustard, jatropha, and groundnut significantly increased the plant growth parameters and total chlorophyll content of tomato and reduced RKN population and galls [23,24]. The saponin released from camellia seed cake can kill muciferous mollusks [25] and could therefore be used to prevent the damage caused by RKNs. Camellia seed cake acts as a non-conventional fertilizer and effectively suppress PPNs [26]. Li et al. [27] reported that methyl thiobutyrate inhibits *Caenorhabditis elegans* and *M. incognita*. The seeds and seed cakes of neem, madhuca, and simarouba are being incorporated into the soil to control PPNs and insects [20,28].

Presently, molecular docking is an excellent technique for creating and providing details about ligand and receptor communications that are supportive of forecasting the binding course of ligands to their aim proteins or DNA [29]. Additionally, this method also helps in systemic learning by delivering a molecule on the binding site of the targeted macromolecule in a non-covalent manner, foremost to a specific binding at every ligand’s active site [30]. Keerthiraj et al. [31] reported the multi-modal inhibitory action of α-bulnesene and α-guaiene against three target proteins, viz., acetyl cholinesterase, odorant response gene-1, and odorant response gene-3. Kundu et al. [32] found that in silico molecular interaction screenings of major essential oil constituents and the seven selected target proteins of the nematode indicated the highest binding affinity of the geraniol–odorant response gene complex due to extensive H-bonding and hydrophobic and π-alkyl interactions. In this study, the nematicidal potential of CSC was investigated to explore a green strategy for managing *M. incognita*. In addition, the nematicidal substances in CSC were also identified using GC-MS analysis. This paper highlights how CSC can be used to manage *M. incognita* to reduce its harmful effects. It also examines potential mechanisms involved in the control of *M. incognita* based on in silico molecular docking analysis.

## 2. Materials and Methods

### 2.1. Materials

The pathogen tested in the experiment was the RKN *Meloidogyne incognita*. However, *M. incognita* egg masses and J2s were obtained from infected brinjal roots. Methanol (HPLC grade) was purchased from Fisher Scientific (Illkirch, France). In addition, cotton seed cakes were obtained from the local market of Aligarh, U.P., India.

### 2.2. Collection and Multiplication of Inoculums (J2s) of M. incognita

Brinjal root infected with *Meloidogyne* spp. was taken from the nearest field around the Aligarh district. However, the identification of *M. incognita* was made by observing perineal pattern characteristics [33]. The J2s of *M. incognita* were multiplied on brinjal and maintained in a greenhouse at the Botany Department, A.M.U., Aligarh, U.P., India. Brinjal plants were uprooted gently so that egg masses were not detached from the root, and then were washed in distilled water properly until the whole soil debris was removed. Egg masses were picked from infected roots using sterilized forceps. The obtained egg masses were washed with DDW and poured into 25 μm pore-size mesh sieves having crossed layers of tissue and placed in petri dishes with water just deep enough to contact the egg masses, which can favor the 2s hatching. These petri dishes were kept in a BOD incubator to hatch J2s of *M. incognita*. The mesh retained the egg masses while the hatched J2s moved through the sieve and sank to the bottom of the petri dishes. The suspension containing hatched J2s was collected daily, and fresh DDW was added. The concentration of fresh-hatched J2s was standardized as per requirement and stored for further study.

## 3. Identification of Meloidogyne Species through Scanning Electron Microscopy (SEM) Analysis

**Figure 1:**
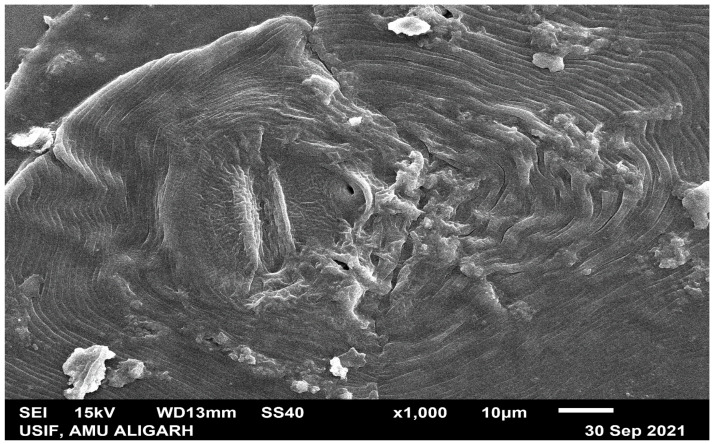
Scanning electron microscopy showing the perineal pattern of *M. incognita*. The high squared dorsal arch and wavy striae are key features of *M. incognita*.

SEM analysis was utilized for the identification of *M. incognita*. An adult female of *M. incognita* was picked up from nematode-infested roots of brinjal, and the perineal pattern was prepared by the technique given by Isabel and Santos [34]. The perineal pattern of nematodes coated with gold (14 nm) was observed, followed by taking images under SEM (JSM-6510 LV Jeol-Japan). The morphological characteristics of the perineal pattern of *M. incognita* were studied for the identification of RKN species with the help of obtained SEM images (Figure 1).

An angularly oval structure with a prominent high dorsal arch in a typical pyriform is one of key characteristics of the perineal pattern, as well as striations in distinct waves which show bending towards lateral lines, straighter with an oval appearance in ventral regions, and not interrupted.

### 3.1. Preparation of CSC Extract 

CSC was crushed with an average diameter of 0.2 mm utilizing a mortar and pestle to form a fine powder. Then, 0.5 g of CSC powder was dissolved in 20 mL of methanol (HPLC grade), the mixture was vortexed and placed in an ultra-sonicator bath for 15 min, and centrifuged at 500 RPM for 15 min. After that, the mixture was processed through filter paper (Whatman filter paper No. 1). Then, the filtered sample was placed into an Eppendorf tube and sent by speed post to the Central Facility Lab, JNU Delhi, for GC-MS analysis. For hatching, mortality, and penetration bioassay, 1 g of CSC was weighed and dissolved in 1000 mL of DDW. This was then filtered through filter paper (Whatman filter paper No. 1). This mixture was named as 1000 mg/L solution (stock solution), and various concentrations (250, 500, and 750 mg/L) were made from this stock solution by the addition of the requisite quantity of DDW.

### 3.2. GC-MS Analysis of CSC Extract

Identification of bioactive compounds present in the methanolic extract of CSC was performed by GC-MS (Shimadzu QP2010 Plus) equipped with an Rtx-5MS capillary column (30 m in length and 0.25 mm each internal diameter and film thickness). A sample with a volume of 250 µL was transferred into vials of GC glass, and out of it 1 µL was subjected to injection in the column with a splitting ratio of 10 @1.21 mL/min using Helium as a carrier gas. The temperature of the oven was fixed at 100 °C, then increased to 250 °C with a 5 °C per minute increment, and lastly to 280 °C with a 10 °C/min increment, while the MS detector was used in full scan mode to observe compounds through distinct peak fragmentation patterns. The data were examined with the GC-MS solutions (Lab Solutions) program, peaks were manually integrated, and chromatograms were deconvoluted with the same software. The retention periods were used to identify the compounds. Various compounds were quantified and determined based on peak regions and molecular masses, respectively. The discovered compounds were validated through a comparison of peak spectrum with that of mass spectra from mentioned library databases as a reference (National Institute of Standards and Technology (NIST 14s, NIST 14, and Wiley 8)). 

### 3.3. Mortality Test

The nematicidal activities of different concentrations (250, 500, 750, and 1000 mg/L) of CSC extract against J2s of *M. incognita* were determined, and LC 50 values were calculated for each treatment. To determine the influence of CSC on the mortality of J2s, 1.5 mL of DDW containing 50 freshly hatched J2s was put into petri plates having 8.5 mL of different CSC concentrations. As a control, the petri plates with only DDW were used. Petri plates were appropriately sheltered using parafilm to prevent evaporation, and mortality of J2s was observed using a dissecting microscope after 12, 24, 36, and 48 h of the exposure. There were five replications for each treatment. The J2s were considered alive if they showed any movement or in a zigzag form [35]. However, dead J2s of nematodes did not show any motion following transfer to tap water when examined by a very sharp needle [36]. The concentration and percent mortality data were subjected to probit analysis, and LC 50 values of all treatments were calculated [37,38]. The percent (%) mortality was determined over control according to the following formula.

(1) $$\mathrm{Percent}\;\mathrm{Mortality}=\frac{C0-T\alpha }{C0}\times 100$$

where the components are the following: 

*C*0 = Number of J2s alive in control;

*Tα* = Number of J2s alive after exposure period of 12, 24, 36, and 48 h in different concentrations of CSC.

### 3.4. Hatching Bioassay

The J2 hatching inhibitory activity of different concentrations (250, 500, 750, and 1000 mg/L) of CSC was determined by the egg mass dipping method. Five egg masses of average size were hand-picked with the help of sterilized forceps from the *M. incognita*-infected roots of brinjal and put into petri plates with 10 mL of CSC in different concentrations, as mentioned above. Petri plates were covered adequately by using parafilm to prevent evaporation and incubated at 28 °C. Five egg masses were placed in petri plates with DDW in the control setup. Each treatment had five replications, except the control. Hatching data were obtained through the counting of the number of J2s that hatched in each replicate after 4 days using a binocular microscope. The percent inhibition of J2 hatching was calculated from mean value by applying the following formula.

$$\mathrm{Percent}\;(\%)\;\mathrm{inhibition}\;\mathrm{in}\;J2s\;\mathrm{hatching}\;=\;\;\frac{No.of\;J2s\;hatched\;in\;control-No.of\;J2s\;hatched\;in\;each\;concentration}{No.\;of\;J2s\;hatched\;in\;control}\times 100\;$$

### 3.5. J2 Infection Bioassay

The penetration rate of J2s in the roots of tomato plants was performed in lab conditions. The seeds of susceptible tomato cultivars were sown in pots in a greenhouse at the Botany Department, A.M.U., Aligarh, to obtain the seedlings. After that, disposable tea cups (7 cm) were filled up with a mixture of washed river sand (40 g) and CSC (10 g) powder. The tea cups were watered regularly for appropriate decay of applied CSC. After decomposition, three-week-old tomato seedlings were transplanted in each disposable tea cup. The established tomato seedlings were treated with 90 J2s/pot and arranged in a randomized complete block design. The tomato plants inoculated with J2s were gently uprooted after 3 and 5 days for observation of the penetration rate. During sampling, the plants’ root systems were thoroughly washed in running tap water to take away attached sand particles. Further, plant roots were kept on paper to remove excess moisture, then they were cut into approximately 2 mm pieces. To find out the J2s’ penetration rate, complete root systems were stained in a solution of fuchsin-lactoglycerol followed by clearing with lactoglycerol [39]. Later, stained roots were put on a glass slide for observation under a stereomicroscope accompanied by counting of penetrated J2s.

### 3.6. Molecular Modeling and Docking

#### Molecular Docking

The AutoDock Vina program [40,41] was utilized to complete molecular docking, which was performed on an Intel (R) Core (TM) i5-4200U CPU-2.10 GHz, 64-bit processor. We investigated the interactions between docked receptors and ligands using Discovery Studio (DS) version 4.1 Client. Based on the docking score and interacting residues, the most chosen docking conformation communications of AChE with three ligands (9,12-Octadecadienoic acid, n-Hexadecanoic acid, and Tetradecanoic acid) were examined. DS Visualizer version 4.1 (Dassault Systèmes BIOVIA, 2017) [42] was used to prepare all interactions, identify the active site residues, and depict 2D poses.

### 3.7. Protein and Ligand Preparation

The NCBI and UNIPROT databases were used to download the hypothetical protein sequences. The molecular and biological functions of the obtained sequences were searched and annotated using BLAST servers (https://blast.ncbi.nlm.nih.gov/Blast.cgi (accessed on 11 December 2022) and https://parasite.wormbase.org//Tools/Blast, (accessed on 11 December 2022)). The PDB database and NCBI Blast program were used to find the template for modeling the secondary structure of sequences. Modeler version 9.24 was used to perform additional homology modeling on the protein AChE. The chemical structures of 9,12-Octadecadienoic acid (CID: 3931), n-Hexadecanoic acid (CID: 985), and Tetradecanoic acid (CID: 11005) were acquired from the PUBCHEM database (https://pubchem.ncbi.nlm.nih.gov, (accessed on 11 December 2022)) as sdf files. Before the molecular docking studies, ligands were minimized using the MM2 force field. The AutoDock Vina Dock prep tool was used to prepare the ligand molecules. The receptor files were appropriately saved in the pdb format, and all prepared ligand files were saved in Mol2 plan.

### 3.8. Statistical Analysis

The collected data on various studied attributes were analyzed statistically using R software (version 2.14.1). Duncan’s multiple range test (DMRT) was applied to calculate significant differences (*p* = 0.05) between studied attributes. ANOVA was conducted using OPSTAT [43]. The LC 50 values of all the treatments were calculated using OPSTAT [43].

## 4. Results and Discussion

### 4.1. GC-MS Analysis

**Figure 2:**
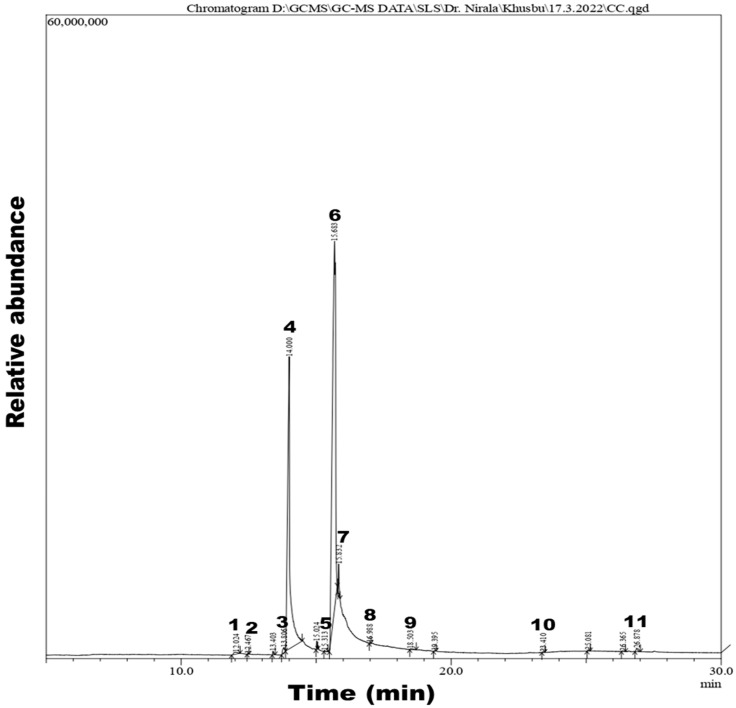
GC-MS chromatograms of methanolic extract of cotton seed cake.

A GC-MS analysis of the methanolic extract of CSC was conducted, and a group of 11 compounds with their retention time, molecular weight and formula, area, and percent area were identified and are depicted in Table 1. The GC-MS chromatogram of the methanolic extract of CSC is shown in Figure 2.

The identified compounds are Tetradecanoic acid (0.41%); Neophytadiene (0.02%); Phthalic acid (0.49%); n-Hexadecanoic acid (36.40%); Methyl stearate (0.06%); 9,12-Octadecadienoic acid (60.45%); Squalene (0.04%); Stigmast-5-en-3-ol (0.27%); Stigmast-5-en-3-ol, (3 beta) (0.07%); Octadecanoic acid (0.14%); 9-Octadecenoic acid (1.12%); and Hexadecanal (0.03%). Of these 11 compounds, n-Hexadecanoic acid (36.40%) and 9,12-Octadecadienoic acid (60.45%) were found as major compounds. Therefore, we conducted molecular docking of these three compounds with the acetyl cholinesterase protein found in *Meloidogyne* spp. All these identified compounds in the CSC extract alone or in combination showed toxicity against J2s and egg masses of *M. incognita*, as shown by our in vitro studies. Of these 11 compounds, n-Hexadecanoic acid (36.40%) and 9,12-Octadecadienoic acid (60.45%) were found as dominant compounds. The GC-MS analysis revealed the presence of above mentioned metabolic active compounds which contributed nematicidal activity against *M. incognita*. Based on studies, some of the constituents shown by GC-MS are biologically active compounds. They were proven to possess nematicidal properties, which may contribute to managing RKNs for sustainable agriculture. GC-MS is a combined analytical technique to determine and identify compounds in a liquid sample. GCMS plays an essential role in the active compound analysis and chemotaxonomic studies of organic materials containing biologically active components.

Our results conform with those of Lu et al. [27], who reported that esters such as methyl stearate and methyl palmitate have been shown to reduce the number of galls and egg masses, suppress the hatching of eggs, and repel larvae and J2s. Some categories of volatile organic chemicals have emerged as having negative impacts on phyto-nematodes, including alcohols (2-octanol and terpineol), phenols, ketones (2-nonanone and 2-undecanone), sulfur compounds (dimethyl disulfide), and aldehydes (decanal and benzeneacetaldehyde) [44,45]. The GC-MS examination of volatile emissions from cotton cake revealed the presence of terpenes, alcohols, sulfur, ketones, and complex combinations of these chemicals in plants, which may serve various purposes, including nematicidal activity [46]. The cotton plant contains terpenes, phenols, proteins, carbohydrates, fatty acids, and lipids as naturally occurring substances [47]. Terpenes are the main class of plant defense compounds produced by cotton plants [48,49]. According to Jibrin et al. [50], cotton seed cake contains flavonoids, alkaloids, saponin, glycosides, and tannins. Qiu et al. [51] reported that cotton seed cakes have linoleic acid, oleic acid, palmitic acid, stearic acid, myristic acid, palmitoleic acid, arachidic acid, and docosanoic acid, among the significant fatty acids. Phenylpropanoids, flavonoids, isoflavonoids, tannins, vanillic acid, caffeic acid, ferulic acid, cinnamic acid, and gallic acid show nematicidal effects [52,53,54].

### 4.2. Molecular Docking Analysis

**Figure 3:**
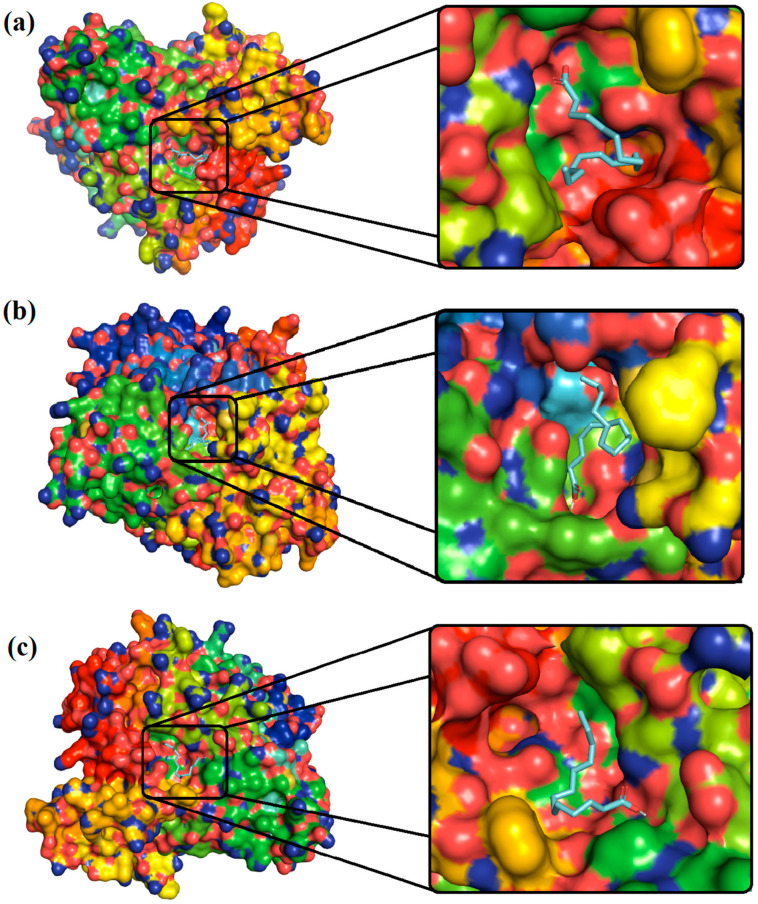
The best docked poses of AChE receptor with (**a**) 9,12-Octadecadienoic acid, (**b**) n-Hexadecanoic acid, and (**c**) Tetradecanoic acid. Blue stick represents the ligands, in which carbon and its valanced hydrogen atoms are shown by blue color, whereas oxygen atoms are displayed by red color. The colors present in protein structure are based on the different amino acids, which exhibit in the protein. The 9,12-Octadecadienoic acid, n-Hexadecanoic acid, and Tetradecanoic acid exhibited high affinity with aromatic and basic amino acids present in the active site of AChE. The components with aromatic rings showed high affinity due to their π–π interactions with organic residues in the binding site of AChE. The most common amino acids involved in binding with ligands are Phe, Arg, His, Gly, Ala, Ser, Leu, and Gln. The amide groups and hydroxyl groups of amino acids are involved in making hydrogen bonds with the -COOH group of these ligands.

**Figure 4:**
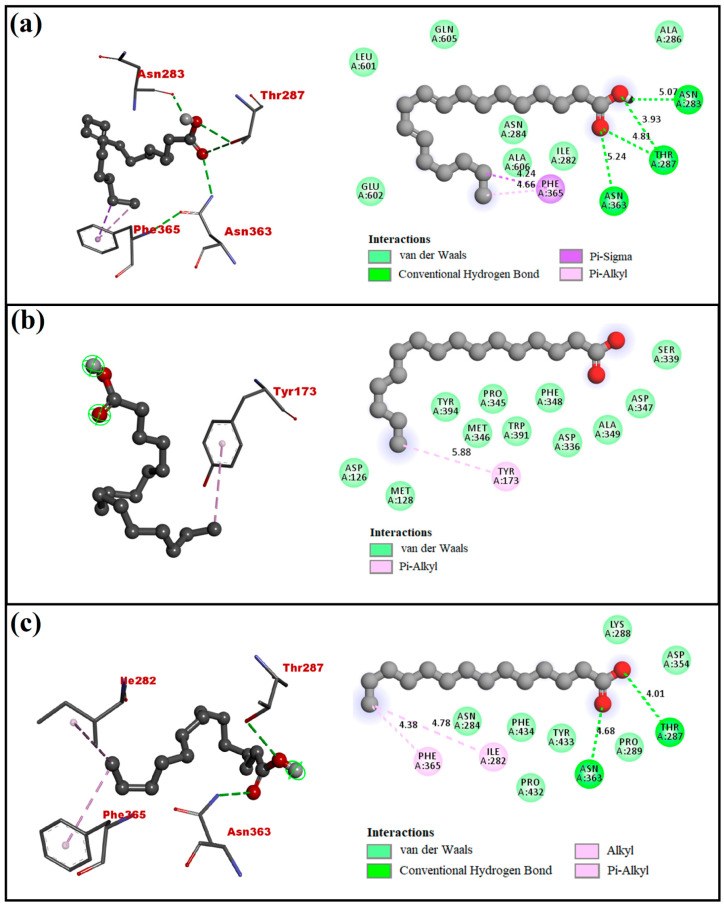
The 2D and 3D representation of interactions for receptor (AChE) docked with (**a**) 9,12-Octadecadienoic acid, (**b**) n-Hexadecanoic acid, and (**c**) Tetradecanoic acid.

Molecular docking analysis was conducted to examine the binding affinities of three compounds, namely 9,12-Octadecadienoic acid, n-Hexadecanoic acid, and Tetradecanoic acid present in CSC extract, to the target protein (AChE) of *M. incognita*. Binding free energy and other docking parameters of interactions between Octadecadienoic acid, n-Hexadecanoic acid, Tetradecanoic acid, and AChE are presented in Table 2. The docked poses of ligands with receptors were predicted and ranked based on the lowest binding free energy evaluated. The best binding conformation or docked pose for studied ligands with target proteins was examined if it displayed the conformation with the lowest overall binding energy. Figure 3 and Figure 4 provide a picture of molecular docking poses for the interaction between ligands and the receptor.

The 3D and 2D representations of interactions between ligands and receptors are depicted in Figure 3 and Figure 4, and other binding details are tabulated in Table 2. It can be seen that 9,12-Octadecadienoic acid with amino acid residues Asn283, Thr287, and Asn363 and Tetradecanoic acid with amino acid residues Thr287 and Asn363 established hydrogen bonds. Additionally, Ile282, Asn284, Ala286, Leu601, Glu602, Gln605, and Ala606 (van der Waals) and Phe365 (Pi-Sigma and Pi-Alkyl) interactions could also be seen in the case of 9,12-Octadecadienoic acid, and n-Hexadecanoic acid interacted with different residues: Asp126, Met128, Asp336, Ser339, Pro345, Met346 Asp347, Phe348, Ala349, Trp391, Tyr394 through van der Waals interactions and with Tyr173 through Pi-Alkyl interactions. Similarly, the residues Asn284, Lys288, Pro289, Asp354, Por432, Tyr433, and Phe434 interacted with Tetradecanoic acid through van der Waals interactions, whereas residue Ile282 via Alkyl and residue Phe365 did so through Pi-Alkyl interaction forces. The binding free energy values are −5.3, −4.5, and −4.9 kcal/mol, observed for 9,12-Octadecadienoic acid, n-Hexadecanoic acid, and Tetradecanoic acid, respectively, suggesting that 9,12-Octadecadienoic acid binds with the receptor (AChE) more efficiently than other two ligands, viz., Hexadecanoic acid and Tetradecanoic acid. Molecular modeling helped identify the tendency of hydrophobic, covalent, and/or non-covalent interactions between receptors and ligands. The ligand efficiency value measured the ability of the molecule to block the target site. Molecular modeling perspectives in sighting the binding affinity of the main three compounds with their target protein helped forecast their solid target binding towards nematode proteins.

Our results are in confirmation with Keerthiraj et al. [31], who demonstrated in their study that molecular modeling and in silico research revealed a multi-modal inhibition of AChE, odorant response gene-1, and odorant response gene-3 by α-bulnesene (−22 to −13 kJ/mol), and α-guaiene (−22 to −12 kJ/mol). Pi-alkyl, pi-sigma, and hydrophobic interactions were the most preferred binding mechanisms against AChE. Thus far, it has been documented that the mode of action of compounds includes disruption and/or change of nematode cell membrane permeability and inhibition of neuro-sensitive receptor proteins such as AChE [55]. PPNs’ neuro-sensitive receptor proteins play a crucial role in neuronal processes and have been widely targeted for chemical inhibition [56]. In this scenario, inhibition of AChE was observed, confirming the simultaneous prevention of nerve impulses [57,58].

### 4.3. Evaluation of Different Concentrations of CSC on J2s’ Mortality and J2s’ Hatching Inhibition of M. incognita

**Figure 5:**
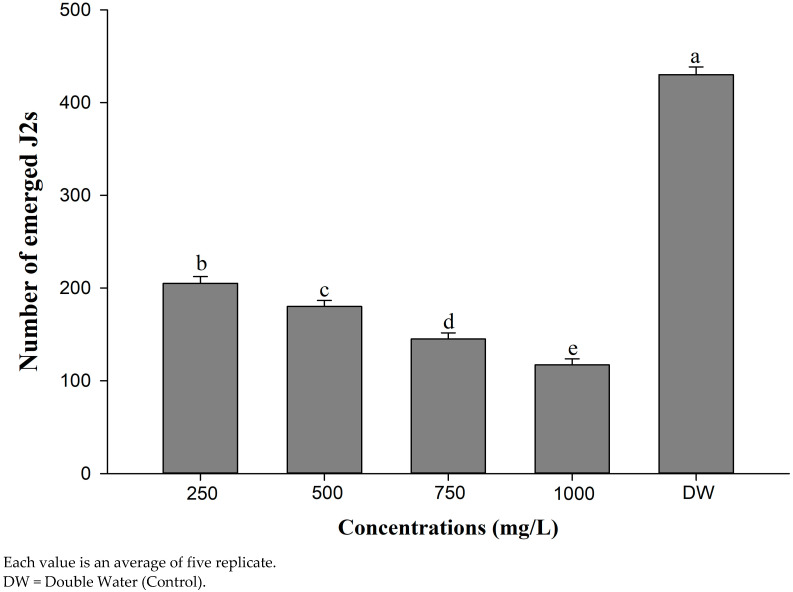
Effect of different concentrations of cotton seed cake on J2 hatching of *M. incognita* over four days of incubation in vitro.

In this bioassay, inhibition of J2 hatches by different concentrations (250, 500, 750, and 1000 mg/L) of CSC extract was analyzed using a direct contact method and a significant difference was reported among the concentrations (Figure 5). All the applied concentrations significantly reduced J2s’ hatching compared to control (DDW). Inhibition of the J2s’ hatching was increased along with an increase in potency of concentrations. However, 250 mg/L also exhibited a significant inhibition of hatching as compared with control. With an increase in the concentration of CSC extract from 250 to 1000 mg/L, there was a corresponding enhancement in inhibition of J2s’ hatching in a successive manner. The highest inhibition of the J2s’ hatch was caused by 1000 mg/L after a 4-day incubation period, followed by 750 mg/L and 500 mg/L. The 250 mg/L was found to be the least prominent in reducing J2s’ hatching. Among all the concentrations tested, 1000 mg/L showed the highest percent inhibition of J2s’ hatching (72.79%). It was followed by 750 mg/L (66.27%) and 500 mg/L (58.13%), whereas 250 mg/L displayed the lowest percent inhibition of J2s’ hatching (52.32%) after 4 days of the exposure period. The individual inhibitory effects of different concentrations (250, 500, 750, and 1000 mg/L) of the CSC extract on J2s’ hatching are given in Figure 5.

The mortality of J2s in different concentrations of CSC extract, viz., 250, 500, 750, and 1000 mg/L, was determined through the direct contact method. Significant differences were observed among the different concentrations (250, 500, 750, and 1000 mg/L). All tested concentrations were found to exhibit some level of toxicity toward J2s of *M. incognita*. Generally, the mortality rates of J2s increased with increasing exposure time and concentrations of the CSC extract. The 1000 mg/L concentration was found to be highly toxic to J2s at a 48 h incubation period, and this toxicity was found significant compared to the other concentrations, viz., 250, 500, and 750 mg/L. Similarly, the incubation period also influenced the percent mortality of J2s significantly and reached the maximum after 48 h of incubation. However, even at 12 h, all concentrations showed a significant difference in mortality compared to the control. There were no deaths of J2s of *M. incognita* reported in DDW (control). The 1000 mg/L concentration was found highly toxic to J2s at all exposure periods (12, 24, 36, and 48 h) compared to the others (250, 500, and 750 mg/L). However, the 250 mg/L concentration also exhibited significant J2 mortality over control (water). All concentrations exhibited higher J2 mortality after 48 h with LC 50-462.95 in comparison with 36 h (LC 50-873.05), 24 h (LC 50-1749.67), and 12 h (LC 50-3362.85) of exposure (Table 3). Our study observed that the lower the LC 50 value of a treatment, the greater its toxicity to J2s of *M. incognita*, and the treatment with a high LC 50 value was the least toxic to J2s. With the increase in concentrations from 250 mg/L to 1000 mg/L and incubation periods from 12 to 48 h, there was an increase in J2 mortality on a successive basis. The results clearly indicate that all concentrations were highly toxic to J2s and caused mortality from 42.00% to 70.00% (Table 4). The individual toxic effects of different concentrations (250, 500, 750, and 1000 mg/L) of CSC on J2 mortality are presented in Table 4.

Our in vitro studies proved that all concentrations, viz., 250, 500, 750, and 1000 mg/L, showed toxicity to J2s and egg masses of *M. incognita*. Various compounds released by the CSC extract were found toxic to J2s and egg masses. This might be due to the presence of multiple compounds in the extract of CSC, which may either affect embryonic development, kill J2s, or dissolve egg masses. Our results conform with those of Lima et al. [59], who reported that volatile compounds formed by the decay of mustard leaf or mustard seed meal applied at the concentrations of 1.6% and 0.8% reduced hatching of J2s of *M. incognita* by 82% and 99.7%, respectively. Through GC-MS, 11 compounds were identified, which alone or in combination show nematicidal potential against *M. incognita*. Among them, 1,2-Octadecadienoic acid, Hexadecanoic acid, and Tetradecanoic acid showed the best nematicidal efficiency against *M. incognita*, as shown by our in silico study. As concentrations and exposure periods increase, mortality of J2s and percent inhibition of J2s’ hatching also increase. In this way, the nematicidal potential of extracts depends upon incubation time and concentrations [60]. The mechanism involved in the suppression of nematodes may include degradation and denaturation of proteins, enzyme functioning inhibition of the respiratory chain’s electron transport, or the phosphorylation of ADP [61]. During the recent decade, inquiries regarding RKN management were focused on tools for egg hatch inhibition factors [62] or the production of secondary metabolites [63,64]. Jardim et al. reported that (E)-cinnamaldehyde volatile caused 100% immobility and 84% mortality of *M. incognita* [65]. Water added to the soil amended with oleaginous cake stimulates the release of volatiles, some with nematicidal activity, such as ammonia, phenols, and aldehydes [66]. The nematicidal activity of neem cake could be due to any of the several known biologically active components, such as nimbine, nimbidine, thionemone, kaempferol, and azidirachtin, which are nematicidal in nature [67]. Glucosinolates, the constituents of rapeseed cake, have nematicidal and nemostatic effects on RKNs due to their hydrolyzed substances such as isothiocyanates [68]. Strong nematicidal effects were demonstrated by coumaric, benzoic, phydroxybenzoic, and nicotinic acids against *M. incognita* [69]. The aqueous extracts of dry and fresh leaves of Eucalyptus citriodora include vanillic, ferulic, caffeic, coumaric, benzoic, chlorogenic, and hydroxybenzoic acids, which have nematicidal effects [35]. All of the compounds studied by Zhang et al. [70], which examined butyric, caprylic, capric, lauric, myristic, palmitic, and oleic acids, dramatically decreased *M. incognita* reproduction. A tactic to lessen the buildup of organic waste in the environment is the use of organic material in agriculture. Aqueous extraction can maximize the utilization of waste while enabling the discovery of nematicidal compounds, which can subsequently be synthesized and used to create natural nematicides that are less harmful to the environment.

### 4.4. Effect of Different Concentrations of CSC on J2s’ Penetration in Roots of Tomato Seedlings

Different concentrations of CSC, viz., 250, 500, 750, and 1000 mg/L, were found to reduce J2s’ penetration in the roots of tomato seedlings to a varying degree as compared to J2s alone inoculated in tomato seedlings after 3 and 5 days of inoculation. The 1000 mg/L concentration showed the highest inhibition of J2 penetration, and 250 mg/L displayed the most negligible inhibition of J2 penetration in the roots of tomato plants. Other concentrations, viz., 500 and 750 mg/L, also showed significant inhibition of J2 penetration. With an increase in concentrations from 250 mg/L to 1000 mg/L, there was an increase in inhibition of J2 penetration. In tomato seedlings inoculated with only J2s, the highest J2 penetration was observed. In general, a higher concentration prevented the penetration of J2s in the tomato roots, whereas the lowest concentration displayed minimum penetration after 3 and 5 days of inoculation compared with the seedlings inoculated with only J2s. From the above results, it was found that the CSC extract has nematostatic and nematicidal properties and could be used for the management of the RKN *M. incognita* to promote a sustainable environment. The inhibition of J2s’ penetration in the roots of tomato plants and the resulting toxicity depends on the exposure period and concentration of the CSC extract. This means that at the highest concentration a lower penetration rate was achieved, and at a lower concentration a higher penetration rate was found. The individual inhibition effects of J2s’ penetration by different concentrations (250, 500, 750, and 1000 mg/L) of CSC are given in Table 5.

From the above results, it was found that CSC has tremendous nematostatic potential as well as nematicidal efficacy, which could be helpful in the sustainable management of RKNs. Our results conform with work conducted by Dammini-Premachandra et al. [71], who reported that repellent properties of the bionematicidal component, which are absorbed by roots from the soil, may have an impact on chickpea infection in roots caused by J2s. The reduction in penetration rate was increased when there was less J2s attractiveness towards plant roots and an insufficiency of root diffusion to fascinate nematodes through bionematicides [72]. The decrease in the population of *M. incognita* is also related to J2s’ entrance into the roots of tomato seedlings or arresting the J2s’ development further, which penetrates roots. Furthermore, bionematicidal compounds absorbed by the plants might be activated by compound reactions that trigger plant resistance mechanisms. The putative method of nematode control was revealed to be both direct and indirect, involving enhanced health of the plant host and variations in root physiology that contribute to increased infection resistance. Immobility and J2s’ mortality of M. incognita were caused by their contact with volatile organic compounds such as neem and mustard, and also decreases in infectivity and reproduction when J2s were inoculated into tomato plants [73]. Ulfa et al. [74] reported that turmeric extract in various solvents significantly inhibited RKN egg hatching and root penetration but had no effect on RKN development or reproduction.

## 5. Conclusions

The aim of the current study is to identify several metabolic compounds from a CSC extract using the GC-MS technique and to evaluate its nematotoxic potential against *M. incognita*. Using the GC-MS technique, 11 active compounds were identified, out of which 9, 12-Octadecadienoic acid, Hexadecanoic acid, and Tetradecanoic acid were found as major compounds. All these identified compounds alone or in combination showed toxicity against *M. incognita*, as shown by our in vitro studies. The presence of several compounds in the CSC extract is an important characteristic, most likely associated with its nematicidal effects, which can be explored for the production of natural nematicides. The CSC extract was effective in controlling *M. incognita*, especially when applied as amendments, and promoting tomato growth. The present investigation also employed a molecular docking tool to relate the nematotoxic potential of bioactive compounds, viz., 9,12-Octadecadienoic acid, Hexadecanoic acid, and Tetradecanoic acid, with the AChE protein of *M. incognita*. In silico results suggested that the three compounds mentioned above have a better binding affinity to the AChE protein of *M. incognita*. The molecular docking technique to understand the bioactivity of the compounds is a novel approach to the logic-driven selection of natural products and the detection of bio-pesticidal leads. In addition to its low cost, CSC is not much used for animal feed, as it contains several metabolic compounds, viz., Tetradecanoic acid, Neophytadiene, Phthalic acid, n-Hexadecanoic acid, 9,12-Octadecadienoic acid, Squalene, Stigmast-5-en-3-ol, Stigmast-5-en-3-ol, and Hexadecanal, etc., thereby constituting a sustainable alternative for nematode control and plant growth. Using indigenous seed cake is a safe, cost-effective, and eco-friendly control option for the management of RKNs under integrated pest management (IPM). As a result, our strategies of using CSC in agricultural approaches may boost the physiochemical properties of the soil and provide a method for controlling RKNs that is cheap and eco-friendly.

## Figures and Tables

**Table 1:** List of constituent compounds present in methanolic extract of cotton seed cake determined by GC-MS analysis.

Peak Number	Retention Time	Area	Area%	Name of Compound	Molecular Weight	Molecular Formula
1	12.024	1,906,993	0.41	Tetradecanoic acid	228.37	C_14_H_28_O_2_
2	12.467	114,218	0.02	Neophytadiene	278.5	C_20_H_38_
3	13.806	2,301,736	0.49	Phthalic acid	166.13	C_8_H_6_O_4_
4	14.000	170,834,132	36.40	n-Hexadecanoic acid	256.42	C_16_H_32_O_2_
5	15.313	282,292	0.06	Methyl stearate	298.5	C_19_H_38_O_2_
6	15.683	283,687,872	60.45	9,12-Octadecadienoic acid	280.4	C_18_H_32_O_2_
7	15.832	5,263,067	1.12	9-Octadecenoic acid	282.5	C_18_H_34_O_2_
8	16.988	139,234	0.03	Hexadecanal	240.42	C_16_H_32_O
9	18.503	640,704	0.14	Octadecanoic acid	284.47	C_18_H_36_O_2_
10	23.410	179,662	0.04	Squalene	410.7	C_30_H_50_
11	26.878	1,276,644	0.27	Stigmast-5-en-3-ol	414.7	C_29_H_50_O

**Table 2:** The interactions of the different ligands with AChE.

Ligands	Binding Free Energy (kcal/mol)	Interactions	
		Hydrogen Bonding	Others
9,12-Octadecadienoic acid	−5.3	Asn283, Thr287, and Asn363	Ile282, Asn284, Ala286, Phe365, Leu601, Glu602, Gln605, and Ala606
n-Hexadecanoic acid	−4.5	-	Asp126, Met128, Tyr173, Asp336, Ser339, Pro345, Met346 Asp347, Phe348, Ala349, Trp391, and Tyr394
Tetradecanoic acid	−4.9	Thr287 and Asn363	Ile282, Asn284, Lys288, Pro289, Asp354, Phe365, Por432, Tyr433, and Phe434

**Table 3:** Nematicidal activity of cotton seed cake against J2s of *M. incognita*.

Treatment	Exposure Period (Hours)	LC 50 Value in mg/L (95% CL)
Cotton seed cake	12	3362.85
	24	1749.67
	36	873.05
	48	462.95

LC 50—lethal concentration caused 50% mortality after 8, 16, and 24 h at 95% confidence limits. CL—confidence limit.

**Table 4:** Effect of different concentrations of cotton seed cake extract on the mortality of J2s of *M. incognita* over 12, 24, 36, and 48 h of incubation in vitro.

Treatment	Concentration (mg/L)	Number of Dead J2s (Mean ± SE) at Different Time Intervals (Hours)			
		12	24	36	48
Cotton seed cake	250	8 ^c^ ± 1.53 (8.89%)	13 ^c^ ± 1.53 (14.44%)	19 ^d^ ± 1.53 (21.11%)	30 ^c^ ± 2.08 (33.33%)
	500	12 ^c^ ± 2.08 (13.33%)	18 ^c^ ± 2.52 (20.00%)	30 ^c^ ± 2.31 (33.33%)	47 ^b^ ± 2.08 (52.22%)
	750	17 ^b^ ± 1.53 (18.89%)	25 ^b^ ± 2.31 (27.78%)	39 ^b^ ± 2.31 (43.33%)	54 ^b^ ± 1.53 (60.00%)
	1000	25 ^a^ ± 2.08 (27.78%)	37 ^a^ ± 1.73 (41.11%)	51 ^a^ ± 3.21 (56.67%)	67 ^a^ ± 2.52 (74.44%)
	DW	0 ^d^ ± 0 (0.00%)	0 ^d^ ± 0 (0.00%)	0 ^e^ ± 0 (0.00%)	0 ^d^ ± 0 (0.00%)
	Df	4	4	4	4
	Sum of Squares	1059.60	2271.60	4556.40	8019.60
	Mean Squares	264.90	567.90	1139.10	2004.90
	F-Calculated	36.79	74.72	81.36	123.75
	Significance	0.00001	0.0000	0.0003	0.0002

Each value is an average of five replicates. DW = Double Water (control). SE—Standard Error. J2s—second-stage juveniles. Values given in parentheses represent percent J2 mortality over control. Values given without parentheses represent number of the dead J2s of *M. incognita* in different concentrations. Values followed by the same letter within a column, are not significantly different according to Duncan’s multiple-range test (*p* ≤ 0.05).

**Table 5:** Effect of different concentrations of cotton seed cake extract on the penetration of J2s of *M. incognita* in the roots of tomato over 3 and 5 days.

Treatment	Concentrations (mg/L)	Number of Penetrated J2s (Mean ± SE) at Different Time Intervals (Days)	
		3	5
Cotton seed cake	250	48 ^a^ ± 2.08 (40.00%)	54 ^a^ ± 2.52 (32.50%)
	500	39 ^b^ ± 2.08 (51.25%)	45 ^b^ ± 2.65 (43.75%)
	750	30 ^c^ ± 2.31 (62.50%)	37 ^bc^ ± 2.08 (53.75%)
	1000	25 ^d^ ± 1.53 (68.75%)	29 ^c^ ± 1.73 (63.75%)
	Df	3	3
	Sum of Squares	927	1034.25
	Mean Squares	309	344.75
	F-Calculated	15.84	18.89
	Significance	0.001	0.0005

Each value is an average of five replicates. SE—Standard Error. J2s—second-stage juveniles. Values given in parentheses represent percent inhibition of J2s’ penetration in the roots of tomato. Values given without parentheses represent number of J2s penetrating the roots of tomato. Values followed by the same letter within a column, are not significantly different according to Duncan’s multiple-range test (*p* ≤ 0.05).

## Data Availability

Not applicable.
